# Phospho-TCTP as a therapeutic target of dihydroartemisinin for aggressive breast cancer cells

**DOI:** 10.18632/oncotarget.2971

**Published:** 2015-03-12

**Authors:** Maria Lucibello, Sara Adanti, Ester Antelmi, Dario Dezi, Stefania Ciafrè, Maria Luisa Carcangiu, Manuela Zonfrillo, Giuseppe Nicotera, Lorenzo Sica, Filippo De Braud, Pasquale Pierimarchi

**Affiliations:** ^1^ Institute of Translational Pharmacology, National Research Council, Rome, Italy; ^2^ Medical Oncology Department, Pathology and Molecular Biology Department, Fondazione IRCCS Istituto Nazionale dei Tumori, Milan, Italy

**Keywords:** Advanced breast cancer, phospho-TCTP, DHA, target therapy, combination therapy

## Abstract

Upregulation of Translationally Controlled Tumor Protein (TCTP) is associated with poorly differentiated aggressive tumors, including breast cancer, but the underlying mechanism(s) are still debated. Here, we show that in breast cancer cell lines TCTP is primarily localized in the nucleus, mostly in the phosphorylated form.

The effects of Dihydroartemisinin (DHA), an anti-malaria agent that binds TCTP, were tested on breast cancer cells. DHA decreases cell proliferation and induces apoptotic cell death by targeting the phosphorylated form of TCTP. Remarkably, DHA enhances the anti-tumor effects of Doxorubicin in triple negative breast cancer cells resulting in an increased level of apoptosis. DHA also synergizes with Trastuzumab, used to treat HER2/neu positive breast cancers, to induce apoptosis of tumor cells.

Finally, we present new clinical data that nuclear phospho-TCTP overexpression in primary breast cancer tissue is associated with high histological grade, increase expression of Ki-67 and with ER-negative breast cancer subtypes. Notably, phospho-TCTP expression levels increase in trastuzumab-resistant breast tumors, suggesting a possible role of phospho-TCTP as a new prognostic marker.

In conclusion, the anti-tumor effect of DHA *in vitro* with conventional chemotherapeutics suggests a novel therapeutic strategy and identifies phospho-TCTP as a new promising target for advanced breast cancer.

## INTRODUCTION

Breast cancer is the most common cancer found in women. Many therapies have been shown to be effective in specific subsets of breast cancer patients. Nevertheless, the progression of this disease towards metastasis is a long-standing problem that requires more selective approaches.

Gene expression analysis of primary breast tumors provided important indications that some genetic properties inherent to the primary tumor might predispose cancer cells towards a more aggressive phenotype [1, 2]. The characterization of these genes could have important, potentially therapeutic, implications for identifying tumors at high risk of progression early on.

However, one limitation of these studies is that some genes or signaling pathways which underlie the molecular traits of an aggressive phenotype might be not oncogenes or mutated in cancer cells, and would therefore need to be identified using a different approach. One example is the Kruppel-like transcription factor KLF4/GKLF whose nuclear localization is a prognostic factor of an aggressive phenotype in the early-stages of infiltrating ductal breast carcinoma [3].

All of these studies suggest that the rational way to improve the current risk classification system at primary diagnosis is to compare and, eventually, combine all the information from genomic, proteomic and immunohistochemical studies with the clinico-pathological factors.

In this context, the characterization of novel biomarkers implicated in tumor progression provides new opportunities for combining therapies with classical chemotherapeutics.

Recently, it has been reported that upregulation of TCTP is correlated with poorly differentiated tumors, including breast cancer [4–7], thus highlighting its potential as biomarker for prognosis. TCTP is a highly conserved protein that exhibits pleiotropic biological functions [8, 9]. Gene knockout studies have revealed that TCTP-deficient mice and TCTP-deficient mutants of Drosophila die in the early stages of embryogenesis suggesting its role in cell survival mechanisms [10, 11]. It has been reported that TCTP has the properties of a tubulin-binding protein [12–14] that dynamically interacts with microtubules during the cell cycle [14]. In addition, it has also been demonstrated that TCTP is a key mitotic target of Polo-like kinase1 (Plk1), a serine/threonine kinase, which plays an important role through TCTP for regulating anaphase progression [15, 16]. When PLK1 phosphorylation sites on TCTP are blocked a dramatic increase in apoptotic cells is observed, suggesting that the completion of mitosis is inhibited [16]. TCTP has also been described as an anti-apoptotic factor [4, 17–19]. We previously demonstrated that TCTP is a survival factor that protects breast cancer cells from oxidative stress-induced cell death. Its inhibition reduces tumor cell growth, re-establishes sensitivity to environmental stress and lowers the apoptotic threshold. We proposed TCTP as a “stress hallmark” that may be exploited as a therapeutic target to decrease the resistance of cancer cells to anticancer therapy [20].

Since drug development is time-consuming and costly [21, 22], we identified a TCTP inhibitor among drugs that had already been tested for toxicity in humans, such as Dihydroartemisinin (DHA). DHA is a metabolite of Artemisinin, the active principle of Artemisia annua. DHA is an anti-malaria agent that also demonstrates anticancer activity [23–25]. It has recently been proven that DHA binds human TCTP and reduces the intracellular TCTP levels via ubiquitination and subsequent proteasome-mediated degradation in many tumor cell lines [26].

The objective of this study was to assess whether TCTP inhibition by DHA may affect the growth of breast cancer with less responsive behavior. In particular, we asked if TCTP or any post-translational modifications of TCTP may be correlated with enhanced tumor aggressiveness in breast cancer cells and if it could be affected by the DHA treatment. We also investigated whether the reduction of TCTP induced by DHA would increase the sensitivity of breast cancer cells to drug treatment. Here we show that the reduction by DHA of phospho-TCTP expression levels is critical in breast cancer cell growth and survival. Moreover, we present new clinical data that high phospho-TCTP levels are correlated with high grade tumors, increase expression of Ki-67 and with ER-negative breast cancer subtypes.

These data suggest that phospho-TCTP expression is correlated with enhanced tumor aggressiveness and provide a novel strategy to target advanced breast cancer.

## RESULTS

### Antitumor efficacy of DHA

The antitumor effect of DHA treatment on breast cancer cell lines and TCTP expression levels was investigated at concentrations of 20 and 50 μM which, when previously tested on human peripheral blood mononuclear cells from healthy donors, had shown no cytotoxicity (data not shown).

**Figure 1:**
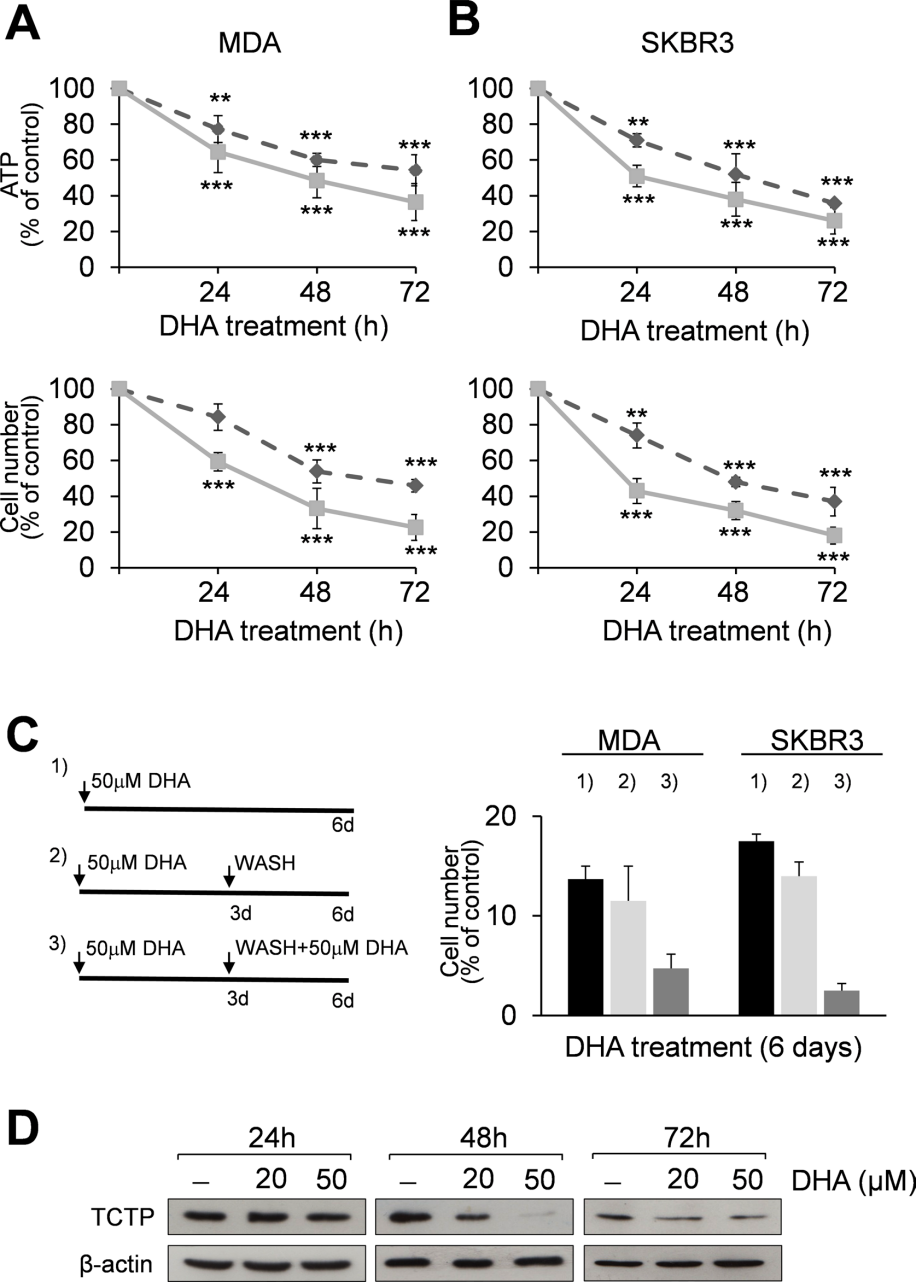
DHA reduces cell viability and TCTP expression levels in MDA and SKBR3 cells MDA **(A)** and SKBR3 cells **(B)** were treated with 20 (----) and 50 μM (–) DHA for 24, 48 and 72 h. At the end of incubation time, the number of viable cells was determined using ATP-assay (upper panels) and trypan blue dye exclusion assay (lower panels). Data are expressed as the percentage of viable cells relative to controls. Values represent the mean ± SD, *n* = 3. Significant differences between treated and control cells, at any time of treatment, are indicated, ** = *p* < 0.01, *** = *p* < 0.001. **(C)** Exponentially growing MDA and SKBR3 cells were cultured for 6 days and treated with 50 μM DHA (panel C, left): 1) cells were exposed to DHA for 6 days; 2) cells were exposed to DHA for 3 days and then the drug was removed; 3) on day 3 cells were washed with fresh media and treated again with 50 μM DHA for 3 days. Data are expressed as the percentage of viable cells relative to controls. Values represent the mean ± SD, *n* = 3. **(D)** Western Blot analysis of TCTP in cell lysates of MDA cells after 24, 48 and 72 h of exposition to DHA. β-actin was used as loading control.

Following the molecular classification for breast carcinoma [27, 28], we chose the MDA-MB-231 cell line with a basal-like phenotype, and the SKBR3, HER2-positive cell line. These cell lines represent *in vitro* models for studying oestrogen receptor (ER)-negative tumors with an aggressive natural history [29, 30]. Exponentially growing MDA-MB-231 (hereafter called MDA) and SKBR3 cells were cultured in the presence or absence of DHA. The number of viable cells, evaluated by ATP (Figures 1A and 1B, upper panels) and trypan blue dye exclusion assays (Figure 1A and 1B, lower panels), decreased severely during the treatment period as compared to untreated cells. Furthermore, a progressive reduction of proliferating cells was observed in cell cultures when exposed to DHA for 6 days. This effect was not reversed when DHA was removed from the cell cultures during the last 3 days. In addition, when the long-term cell cultures (6-days) received a second dose of DHA at day 3, a further reduction in cell viability was observed at day 6, confirming the sensitivity of both cell lines to DHA treatment (Figure 1C).

We then investigated the effect of DHA on TCTP mRNA and protein expression. RT-PCR analysis showed that mRNA levels were unaffected in MDA treated cells (1.38 ± 0.41 and 2.33 ± 0.73 mRNA fold increase versus control cells at 20 and 50 μM DHA respectively; data not shown).

In contrast, TCTP protein levels were almost unchanged at 24 h, but were greatly reduced in MDA cells treated for 48 h with 50 μM DHA (Figure 1D), indicating the inhibitory effect of DHA on TCTP protein expression levels, as previously reported [26, 31]. However, a slight increase of TCTP levels was observed after 72 h, likely due to the DHA short half-life as reported by *in vivo* [32] and *in vitro* studies [33, 34] which suggest that DHA may cause severe damage during the first hours of exposure in breast cancer cells. Similar results were also obtained in SKBR3 cells treated with 50 μM DHA (Figure S1B–C).

### DHA induces a strong reduction of phospho-TCTP levels

Since we did not observe any remarkable reduction of TCTP expression levels during the first 24 h of treatment, when DHA was already highly effective on cell viability, we asked whether any post-translational modifications of TCTP might be affected by the DHA treatment.

**Figure 2:**
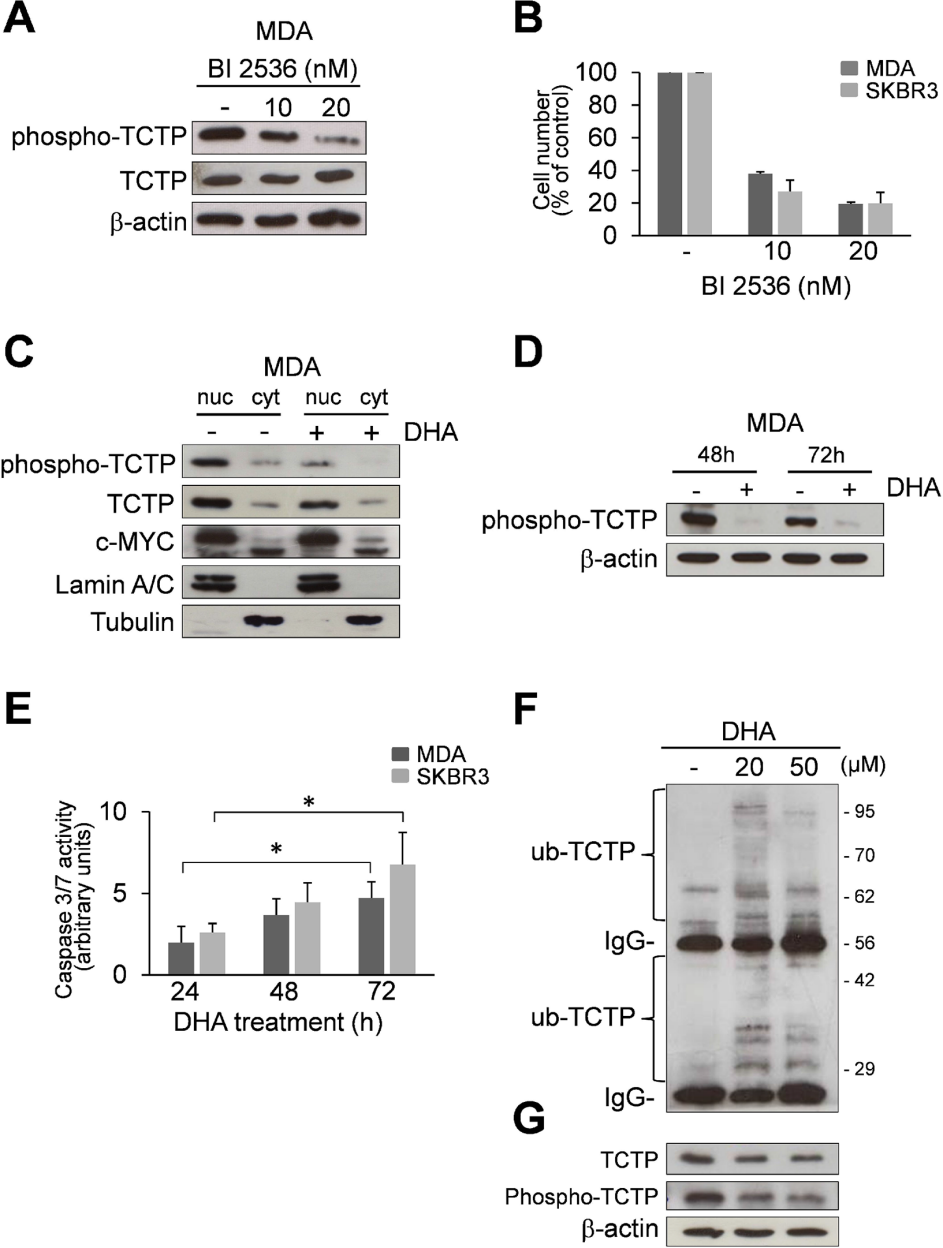
DHA reduces phospho-TCTP levels **(A)** Western Blot analysis of indicated proteins in cell lysates of MDA cells treated with 10 and 20 nM BI 2536 for 48 h. β-actin was used as loading control. **(B)** MDA and SKBR3 cells were treated with 10 and 20 nM of BI 2536 for 48 h. At the end of incubation time, the number of viable cells was determined by trypan blue dye exclusion assay. Data are expressed as the percentage of viable cells relative to controls. Values represent the mean ± SD, *n* = 3. **(C)** Subcellular localization of indicated proteins in MDA cells. MDA cells were treated with 50 μM DHA for 24 h. Nuclear and cytoplasmic fractions of untreated and treated cells were analyzed by Western Blot analysis for the indicated proteins. Lamin A/C and Tubulin were used as loading controls for nuclear and cytoplasmic fractions, respectively. **(D)** Western Blot analysis of indicated proteins in MDA cells after 48 and 72 h of exposition to 50 μM of DHA. β-actin was used as loading control. **(E)** Caspase-3/7 activity (normalized to cell number) was evaluated as a marker of apoptosis in MDA and SKBR3 cells after 24, 48 and 72 h of exposition to 50 μM of DHA. Data are expressed as arbitrary units. Values represent the mean ± SD, *n* = 3. * = *p* < 0.05. **(F)** MDA cells were treated for 24 h with two different concentrations of DHA (20 and 50 μM). Cell lysates (1 mg protein) were immunoprecipitated with a polyclonal anti-ubiquitin antibody, followed by immunoBlotting with an anti-TCTP antibody. **(G)** Cell lysate (10 μg) were analyzed by Western Blot analysis for the indicated proteins. β-actin was used as lysate input loading control (lower panel).

Recent studies have demonstrated that TCTP is an important downstream signalling component of Polo-like Kinase 1 (PLK1); moreover, phosphorylation of TCTP by PLK1 promotes its localization in the nucleus [15, 16]. As shown in Figure 2A and Figure S1A, TCTP is phosphorylated in both MDA and SKBR3 cells. Phospho-TCTP expression levels were reduced by treatment with BI 2536, a selective PLK1 inhibitor [35, 36], confirming that TCTP is phosphorylated by PLK1 in mammary carcinoma cells. The reduction of phospho-TCTP expression levels was also correlated with the inhibition of cell viability (Figure 2B), suggesting that TCTP phosphorylation by PLK1 is a critical event in the regulation of cell growth.

In order to investigate the localization of phospho-TCTP, we carried out a sub-cellular localization analysis. Figure 2C and Figure S1B show that the TCTP was primarily localized in the nucleus, mostly in the phosphorylated form. When cells were treated with 50 μM DHA for 24 h, we observed a dramatic reduction of phospho-TCTP expression levels. In addition, the reduction of phospho-TCTP expression levels was maintained at 48 and 72 h in both cell lines (Figure 2D and Figure S1C).

Since it has been reported that the reduction of phospho-TCTP activity leads to a mitotic catastrophe and cell death [16] we studied the activation of caspases 3/7 in DHA-treated cells. The results showed a significant increase of caspases 3/7 activity in DHA-treated cells (Figure 2E) consistent with apoptotic cell death.

A previous study demonstrated that DHA irreversibly down-regulates c-Myc protein in various human tumor cell lines [37]. As shown in Figure 2C and in Figure S1B, DHA had no notable effect on c-Myc expression levels during the first 24 h of treatment. This suggests that the cellular response to DHA was not related to c-Myc protein expression levels but rather, was strictly associated with the reduction of phospho-TCTP expression levels.

The relevance of phospho-TCTP expression was also investigated in MCF7 and in BT-474, two human breast cancer cell lines, which closely resemble the luminal A and luminal B oestrogen receptor (ER)-positive tumors, respectively [29, 30]. Western Blot analysis of the nuclear and cytoplasmic fractions showed a lower expression of phospho-TCTP in BT-474 cells compared to MCF7 cells (Figure S2A). As the BT474 cell line is derived from a primary invasive ductal carcinoma, while the MCF7 cell line is derived from a metastatic invasive ductal carcinoma, these data strongly suggest that phospho-TCTP expression levels may be associated with enhanced tumor aggressiveness. Phospho-TCTP and TCTP expression levels were dramatically reduced (Figure S2B) and cell proliferation was decreased, in a dose-dependent manner, after 72 h of DHA treatment (Figure S2C). Moreover, DHA induced a caspase-dependent apoptosis as indicated by activation of the caspases 3 and 7 (Figure S2D).

We then investigated whether the ubiquitin-proteasome pathway may regulate the turnover of TCTP in MDA cells during the first 24 h of exposure to DHA. As shown in Figure 2F, DHA induced the ubiquitination and subsequent degradation of TCTP, as previously reported [26]. This suggest that the ubiquitineted-TCTP proteins may not be recognized by PLK1, therefore, causing a reduction in phopho-TCTP levels (Figure 2G).

### TCTP knock down in MDA cells enhances DHA cytotoxicity

To further test the cytotoxicity of DHA in tumor cells with low TCTP expression levels, we knocked down TCTP gene expression in MDA cells using RNA interference.

**Figure 3:**
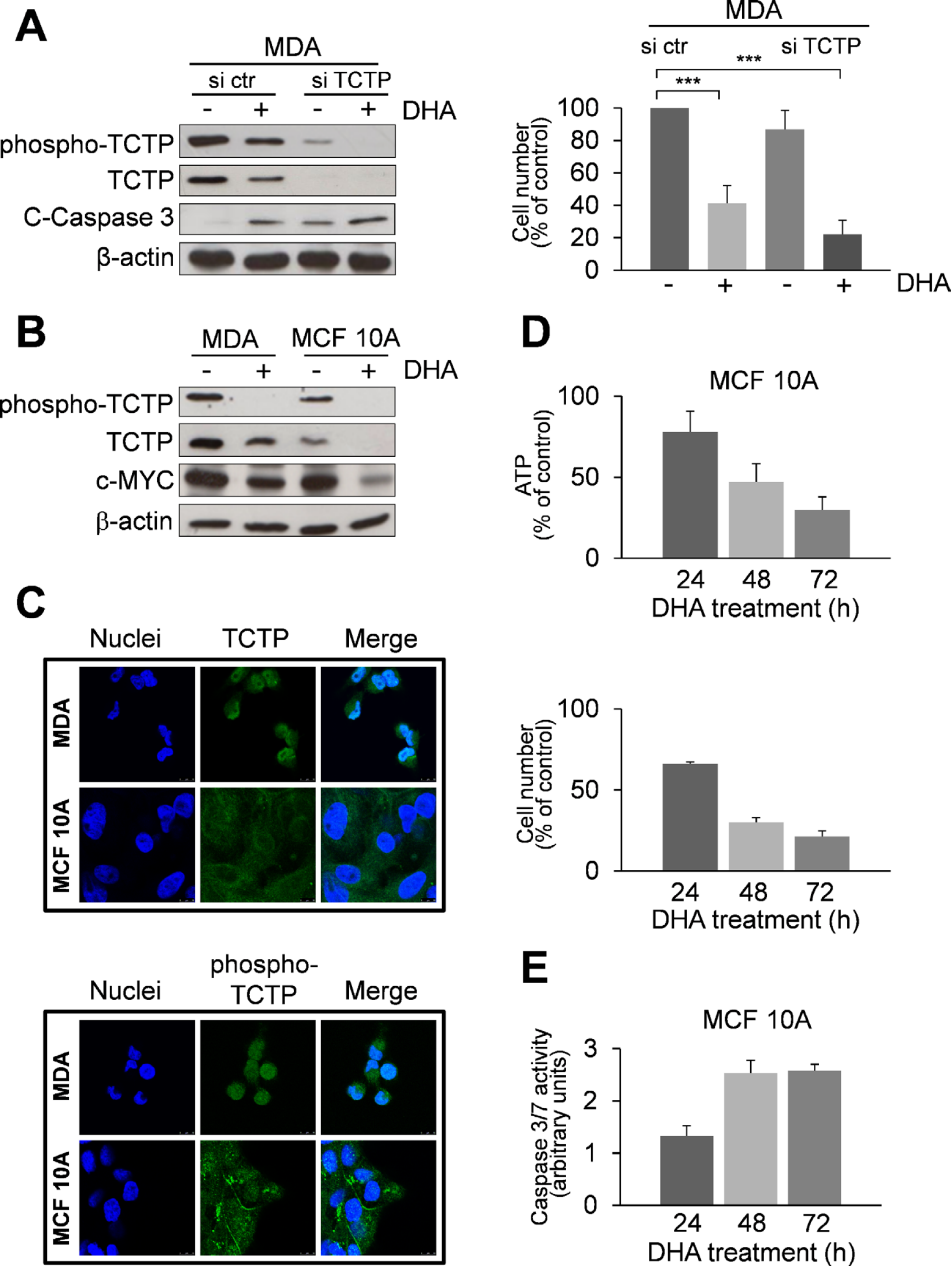
Effect of DHA in TCTP-silenced MDA cells and in the non-tumorigenic human mammary cell line MCF 10A with low TCTP expression levels **(A)** MDA cells were transfected with a scrambled siRNAs (si ctr) or with siRNAs targeting TCTP (si TCTP). Cells were treated with 50 μM of DHA for 48 h. Western Blot analysis of indicated proteins. β-actin was used as loading control (left panel). The number of viable cells was determined by trypan blue dye exclusion assay at the end of incubation time (right panel). Data are expressed as the percentage of viable cells relative to controls. Values represent the mean ± SD, *n* = 4. *** = *p* < 0.001. **(B)** Western Blot analysis of indicated proteins in MDA and MCF 10A cells after 48 h of exposition to 50 μM DHA. β-actin was used as loading control. **(C)** Immunofluorescence detection of TCTP. Cells were analysed by immunofluorescence. Nuclei were stained with 4′,6-diamidino-2-phenylindole (DAPI). TCTP and phospho-TCTP was detected with specific antibodies. The overlay of the two fluochromes is shown (Merge). Images were captured with a LEICA Confocal Laser Scanning Microscopy TCS SP5 system, equipped with a 40x oil immersion objective. Bar = 10 μm. Data from a representative experiment of two with similar results are shown. **(D)** Cytotoxicity of DHA in MCF 10A cells. Cells were treated with 50 μM DHA for 24, 48 and 72 h. At the end of incubation time, the number of viable cells was determined using ATP-assay (upper panel) and trypan blue exclusion assay (lower panel). Data are expressed as the percentage of viable cells relative to controls. Values represent the mean ± SD, *n* = 3. **(E)** Caspase-3/7 activity (normalized to cell number) was evaluated as a marker of apoptosis in MCF 10A cells. Cells were treated as described in the legend 3D. Data are expressed as arbitrary units. Values represent the mean ± SD, *n* = 3.

The expression of TCTP was substantially reduced by specific siRNA as compared to scrambles siRNA (Figure 3A, left panel). Remarkably, in TCTP silenced cells, we observed enhanced cytotoxicity of DHA after 48 h as indicated by cell viability assay (Figure 3A, right panel) and by increase in activated caspase 3 levels (Figure 3A, left panel), hence indicating that additional mechanisms, such as oxidative stress [24, 38], may also mediate cytotoxicity of DHA.

### DHA shows low cytotoxicity in the non-tumorigenic human mammary cell line MCF 10A

We further investigated the cytotoxicity of DHA in MCF 10A, a human breast epithelial cell line, which expresses relatively low phospho-TCTP and TCTP levels in comparison to MDA cells, as shown by Western Blot analysis (Figure 3B) and immunofluorescence staining (Figure 3C). DHA reduced both phospho-TCTP and TCTP expression levels (Figure 3B) and cell viability (Figure 3D). However, DHA exerted a lower cytotoxicity in MCF 10A compared to MDA cells, as indicated by the level of caspase 3/7 activation (Figure 3E). Interestingly, c-Myc levels were greatly reduced in MCF 10A-treated cells (Figure 3B).

### Overexpression of phospho-TCTP rescues TCTP knockdown phenotype

**Figure 4:**
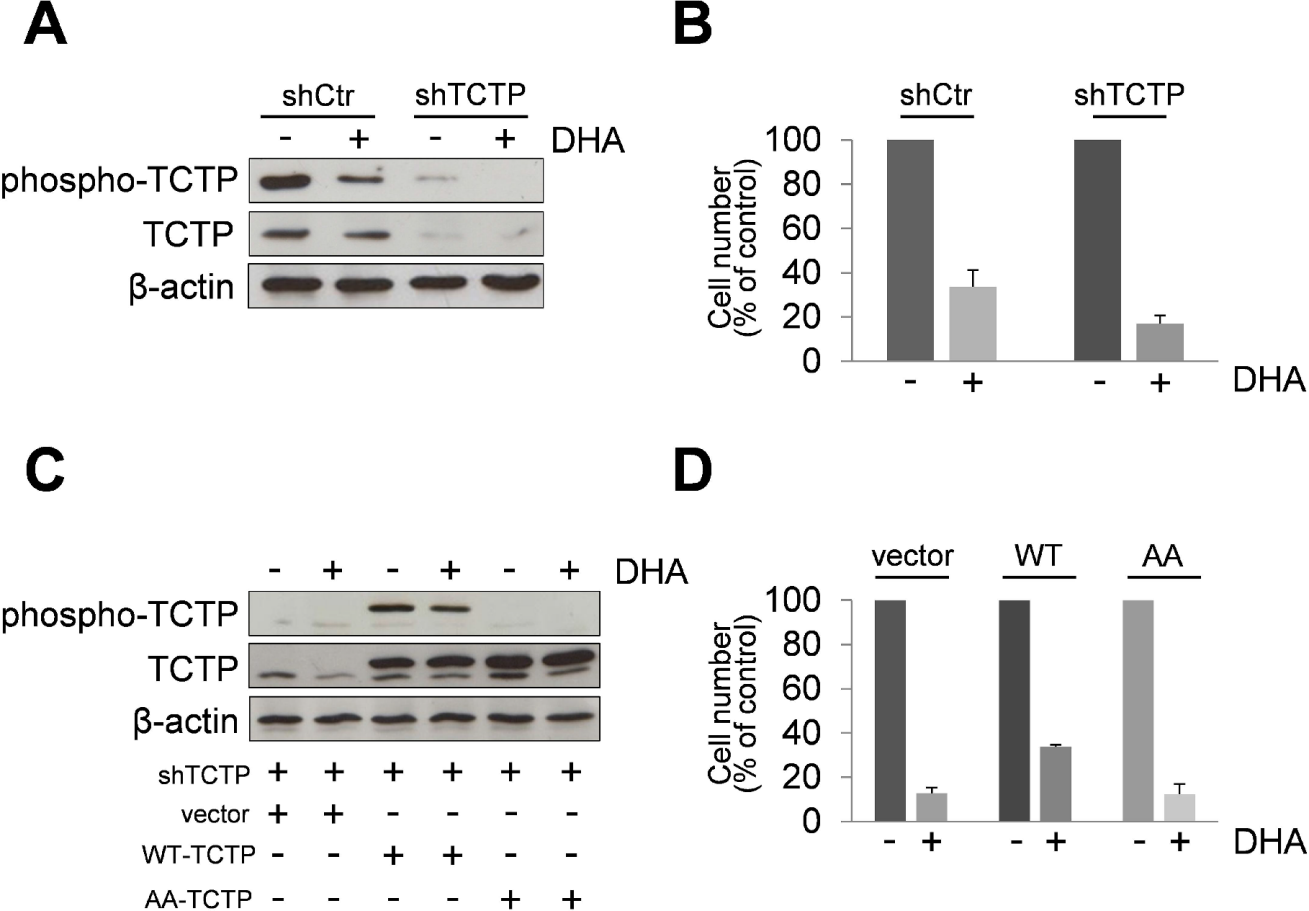
Overexpression of TCTP rescues TCTP knockdown phenotype in HeLa cells **(A)** HeLa cells expressing TCTP-shRNA plasmids (shTCTP) or control-shRNA plasmids were treated for 24 h with 50 μM of DHA. Western Blot analysis of indicated proteins. β-actin was used as loading control. Results from untreated HeLa cells and HeLa ctr-shRNA gave superimposable results and are indicated as control HeLa cells (shCtr). **(B)** Cells were treated with 50 μM of DHA for 24 h. The number of viable cells was determined by trypan blue dye exclusion assay at the end of incubation time. Data are expressed as the percentage of viable cells relative to controls. Values represent the mean ± SD, *n* = 3. **(C)** TCTP silenced-cells were transiently transfected with a plasmid encoding Flag tagged wild-type TCTP (WT) or a mutant lacking two serine residues 46 and 64 (AA). Mock-transfected cells were used as control (vector). After 24 h, cells were treated with DHA (50 μM). A Western Blot analysis was carried out after 24 h of exposure to DHA. **(D)** Cells were treated as in legend 4C. The number of viable cells was determined by trypan blue dye exclusion assay at the end of incubation time. Data are expressed as the percentage of viable cells relative to controls. Values represent the mean ± SD, *n* = 3.

We then investigated the ability of phospho-TCTP to rescue the TCTP knockdown phenotype. We used the easy-to-transfect human HeLa cell line as model for gain-and-loss-of-function experiments. First, we generated TCTP knockdown HeLa cells (Figure 4A). These cells were more sensitive to DHA treatment compared to control cells as indicated by cell viability assay (Figure 4B). Then, TCTP silenced-cells were transiently transfected with a plasmid encoding Flag tagged wild-type TCTP (WT) or a mutant lacking two serine residues 46 and 64 (AA). As shown in Figure 4C, TCTP-silenced cells transfected with the WT-TCTP or with the AA-TCTP constructs expressed high levels of TCTP proteins, indicating that the TCTP-specific shRNA stably expressed in TCTP-silenced cells was able to suppress the basal level of endogenous TCTP, but was unable to prevent exogenous protein overexpression. The exogenous Flag-tagged WT-TCTP or AA-TCTP levels were not reduced by DHA treatment. However, only the overexpression of WT-TCTP was able to rescue cells from the cytotoxic effect of DHA, while AA-TCTP overexpression cannot rescue the TCTP knockdown phenotype, as indicated by cells viability assay (Figure 4D).

### DHA enhances sensitivity to chemotherapy

**Figure 5:**
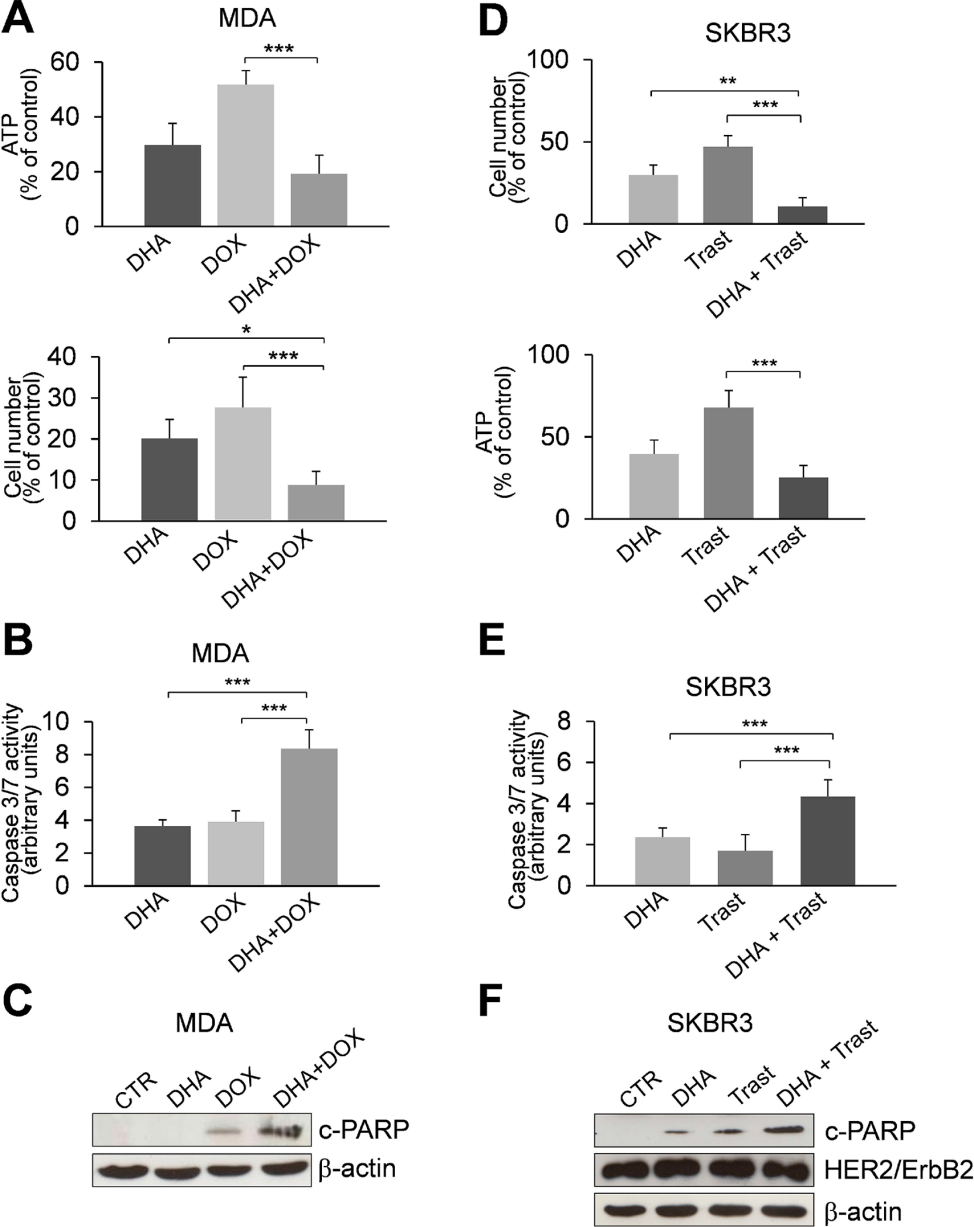
The pharmacological inhibition of TCTP by DHA enhances the sensitivity to chemotherapy **(A)** MDA cells were treated with 50 μM DHA for 24 h. Cell viability was measured after an additional 48 h treatment with 1 μM Doxorubicin (DOX). At the end of incubation time, the number of viable cells was determined by ATP-assay (upper panel) and trypan blue dye exclusion assay (lower panel). Data are expressed as the percentage of viable cells relative to controls. Values represent the mean ± SD, *n* = 4. * = *p* < 0.05, *** = *p* < 0.001. **(B)** Caspase-3/7 activity (normalized to cell number) was evaluated as a marker of apoptosis. Cells were treated as described in the legend 5C. Data are expressed as arbitrary units. Values represent the mean ± SD, *n* = 4. *** = *p* < 0.001. **(C)** Western Blot analysis of indicated proteins in MDA cells. Cell were treated as described in the legend 5A. **(D)** SKBR3 cells were exposed to DHA (20 μM) and were then treated with 50 μg/ml of Trastuzumab (Trast) for 6 days. At the end of incubation time, the number of viable cells was determined by trypan blue dye exclusion assay (upper panel) and ATP-assay (lower panel). Data are expressed as the percentage of viable cells relative to controls. Values represent the mean ± SD, *n* = 5. ** = *p* < 0.01, *** = *p* < 0.001. **(E)** Caspase-3/7 activity (normalized to cell number) was evaluated as a marker of apoptosis. Cells were treated as described in the legend 5D. Data are expressed as arbitrary units. Values represent the mean ± SD, *n* = 5. *** = *p* < 0.001. **(F)** Western Blot analysis of indicated proteins in SKBR3 cells exposed to DHA (20 μM) and then treated with Trastuzumab for 6 days. β-actin was used as loading control.

In order to determine whether the inhibition of phospho-TCTP activity may enhance the sensitivity of breast cancer cells to anti-cancer drugs, we studied the effect of therapeutic agents in MDA and SKBR3 cell lines pre-treated with 50 μM DHA for 24 h. Cell viability was measured after a subsequent 48 h treatment with three different chemotherapy drugs: Doxorubicin, Cisplatin and Paclitaxel. When combined with DHA, Doxorubicin (5 μM) and Cisplatin (50 μM) were more effective in inhibiting the growth of MDA cells compared to the effect induced by each agent alone (Figure S3A). No additional effect was observed by adding Paclitaxel to DHA treatment (data not shown). Notably, the combination of Doxorubicin with DHA showed a great inhibition of cell proliferation even at lower doses of Doxorubicin (1 μM; Figure 5A) and induced a significant increase of apoptosis (Figures 5B and 5C). This drug combination in MDA cells resulted in synergistic effect with CI of 0.661 ± 0.33 (Table S1).

Similar results were obtained when SKBR3 cells were subjected to the same drug treatments (Figure S3B); however, the DHA-combinatorial approach was not efficacious at 1 μM Doxorubicin (data not shown).

We then investigated the effect of Trastuzumab in combination with DHA in SKBR3 cells. Trastuzumab (Herceptin) is a mAb widely used for HER2-positive breast cancers. Following the protocol described in legend 5A, we observed only a slight decrease of cell viability by combining of DHA and Trastuzumab (Figures S4A and S4B). Strikingly, a strong inhibitory effect was observed when cells were exposed to a lower concentration of DHA (20 μM) and then treated with Trastuzumab for 6 days (Figure 5D). Moreover, the combination of DHA with Trastuzumab induced a significant increase of apoptosis as evaluated by the activation of caspase 3/7 (Figure 5E) and by the amount of cleaved- PARP (c-PARP, Figure 5F). The CI value for this drug combination was 1.070 ± 0.2, indicating an additive effect of the two drugs (Table S2). In addition, the expression of HER2 was not affected by DHA suggesting that this drug does not alter the sensitivity of HER2 positive cells to mAb therapy (Figure 5F).

### Correlation between high phospho-TCTP expression and high grade breast tumors

To study the clinical relevance of phospho-TCTP expression in human mammary carcinoma, we performed an immunohistochemistry analysis of phospho-TCTP in 85 human breast cancer specimens.

The association between the clinicopathological characteristics and phospho-TCTP expression is shown in Table 1. Phospho-TCTP expression was not correlated with the patient's age or tumor size (*p* > 0.05) but it was positively associated with the pathological grade (*p* < 0.005). In all grade I cases phospho-TCTP expression was always ≤ 1%, while in grade II or III tumors phospho-TCTP expression was always > 1%. Interestingly, only 5% of grade II or III cases showed a low phospho-TCTP level (1–3%). High phospho-TCTP expression was found in ER-negative breast cancer subtypes which often have a worse prognosis (*p* = 0.022). Moreover, it was also positively associated with cell proliferation as indicated by the increase in Ki-67 (*p* = 0.019), lymph node (*p* = 0.032) and HER2 status (*p* = 0.021).

**Table 1:** Correlation between Phospho-TCTP expression and clinicopathological characteristics in breast tumors

Variable	phospho-TCTP expression				*p* Value
	< 1%	1–3%	4–9%	≥ 10	
	n(%)	n(%)	n(%)	n(%)	
Age					NS(0.96)
≤ 49	2(2)	2(2)	18(21)	6(7)	
50–65	2(2)	4(5)	25(29)	12(14)	
≥ 66	1(1)	2(2)	7(8)	4(5)	
Grade					< 0.005
I	5(6)				
II		4(5)	19(22)	4(5)	
III		4(5)	31(36)	18(21)	
Lymph node					0.032
Negative	5(6)	4(5)	32(38)	8(9)	
Positive		4(5)	18(21)	14(16)	
Tumor size (cm)					NS(0.16)
≤ 2	5(6)	5(6)	37(44)	12(14)	
> 2		3(3)	13(15)	10(12)	
ER+/PgR+	2(2)	4(5)	9(11)	1(1)	0.022
ER+/PgR−	3(4)	3(4)	10(12)	4(5)	
ER−/PgR+			3(4)	1(1)	
ER−/PgR−		1(1)	28(33)	16(19)	
Ki-67					0.019
Negative	5(6)	3(4)	12(14)	5(6)	
Positive		5(6)	38(45)	17(20)	
HER2					0.021
Negative	4(5)	4(5)	21(25)	2(2)	
2+	1(1)	2(2)	7(8)	4(5)	
3+		2(2)	22(26)	16(19)	

Abbreviations: ER, oestrogen receptor; PgR, progesterone receptor; NS non-significant.

**Figure 6:**
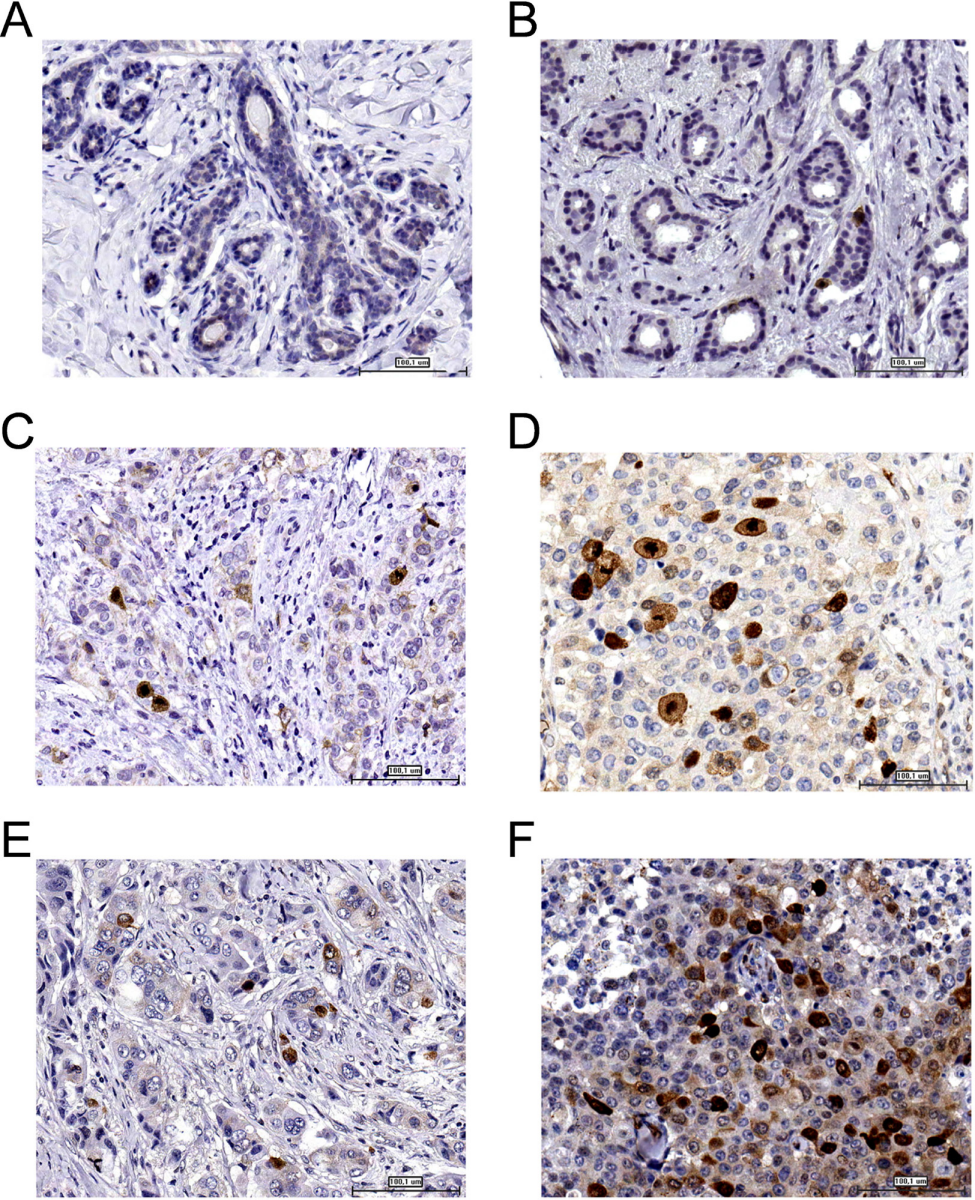
Phospho-TCTP expression is associated with high grade tumors Representative images of the immunohistochemical staining of phospho-TCTP in breast cancer samples. **(A)** Normal human breast tissue. **(B)** Grade G1. **(C)** Grade G2. **(D)** Grade G3. **(E)** Positive response to trastuzumab therapy. **(F)** Negative response to trastuzumab therapy. Image were captured using a 20x objective. Bar = 100 μm

High phospho-TCTP levels were observed only in breast cancer tissues (Figure 6B–6F) as compared to the adjacent normal breast tissues (Figure 6A). In addition, we found a predominantly nuclear staining of phospho-TCTP in high-grade tumors (grades II or III) consistent with the *in vitro* data (Figure 6C and 6D). Notably, among 35 patients treated with trastuzumab therapy we observed high expression of phospho-TCTP (> 10%) when tumors showed resistance to trastuzumab therapy (*p* < 0.005) (Figure 6E and 6F, and Table 2).

**Table 2:** Correlation between Phospho-TCTP expression and response to Trastuzumab therapy

Variable	phospho-TCTP expression			*p* Value
	1–3%	4–9%	≥ 10	
	n(%)	n(%)	n(%)	
Response Trastuzumab-Therapy				< 0.005
−		1(3)	15(43)	
+	3(9)	12(34)	4(11)	

## DISCUSSION

We previously reported that the expression of TCTP in tumor cells is linked to stress-response mechanisms and demonstrated that the reduction of TCTP protein expression is critical for tumor growth/survival. Thus we proposed TCTP as a “stress hallmark” that may be exploited as a therapeutic target to decrease the resistance of tumor cells to therapy [20].

DHA, commonly used in malaria therapy, also shows anticancer activity [23–25]. The mechanism of DHA in tumor cells is still unclear. It has been proposed that highly reactive endoperoxide moiety is at least partially responsible for its anticancer activity [24, 38]. DHA's primary activator involves an iron source, in the form of Fe+2 or heme, or both. The iron(II)-mediated ROS and/or carbon centered radicals can cause cell injury and death [24, 38–40]. Remarkably, the intracellular iron metabolism is altered in breast cancer cells, thereby making them more vulnerable to the cytotoxic effect of DHA [41].

Microarrray analysis reveals that many genes involved in the regulation of cell proliferation or survival are correlated with the cellular response to DHA [24]. However, it remains unclear whether the antitumor activity of DHA may be mediated by specific molecular targets: TCTP is one of the proposed targets for tumor cells [26, 31] and plasmodia. [42–44]

Here, we provide evidence that in exponentially growing human breast cancer cells, with more aggressive phenotypes, phospho-TCTP is located mainly in the nucleus of the cells. The phospho-TCTP expression level is strongly reduced by DHA-treatment and is correlated with the cytotoxicity of DHA. The role of phospho-TCTP activity in growing cells is supported by the reduction of phospho-TCTP caused by BI 2536, a specific PLK1 inhibitor [35, 36]. PLK1 is a crucial serin/treonin kinase in regulating cell proliferation, whose overexpression has been observed in many tumors, including breast cancer [45]. It has been shown that PLK1 phosphorylates TCTP from the G2 phase to the telophase of the cell cycle, and promotes phospho-TCTP nuclear and perichromosomal localization both *in vitro* [15, 16] and *in vivo* (xenograft tumors) [15]. In addition, phospho-TCTP detaches from the spindle at the metaphase-to-anaphase transition [16]. Since the dynamics of spindle microtubules appear to be critical for spindle function, the reduction of phospho-TCTP may induce a disturbance of microtubule regulation with critical consequences for cell fate. Thus, we speculate that nuclear overexpression of phospho-TCTP may be required for proper anaphase progression in breast cancer cells. Our data suggest that the inhibition of phospho-TCTP activity by DHA can mimic spindle poisons in high proliferating breast cancer cells, and may reduce the side effects of this class of therapeutics [46].

In this context, the reduction of phospho-TCTP levels by DHA may represent a critical point in a sequence of events leading to apoptosis cell death. However, we cannot exclude that the antitumor effects of DHA may also be mediated by a more general mechanism through generation of ROS. In DHA-treated cells large reduction of the whole TCTP content by a 48 h treatment may also enhance DHA cytotoxicity, in accordance with our previously reported findings showing that TCTP is a critical survival factor that protects breast cancer cells from oxidative stress [20]. Consistent with this data, we observe a greater reduction of proliferating cells when TCTP-silenced cells were treated with DHA. Conversely, enforced overexpression of phopho-TCTP rescued DHA toxicity in TCTP-silenced HeLa cells.

Remarkably, we observed that MCF 10A cells were more protected by the DHA-induced cytotoxicity compared to MDA cells as suggested by the lower activation of caspase-3 and caspase-7. As MCF10A cells contain wild-type p53 [30], we can speculate an association between p53 activation and down regulation of c-Myc expression levels as a cellular response to the cytotoxicity of DHA. In contrast, MDA cells have a different molecular phenotype and express mutants of p53 [30, 47] and undergo apoptosis in response to damage induced by DHA. Therefore, DHA cytotoxicity is enhanced in breast cancer cells with mutated p53, i.e. in the more aggressive and therapeutically less responsive breast tumors [48].

Our data also show that TCTP and phospo-TCTP are present at the cell membrane. In our previous work we showed that TCTP-silenced MDA-MB-231 cells display a different morphology compared to control cells, suggesting that TCTP may play a critical role in cell shaping [20]. Consistent with this finding, it has been reported that TCTP possesses an actin-binding sites [12], moreover, Baziel et al. have proposed a critical cytoskeleton-mediated cellular function for TCTP [13]. Altogether, these findings suggest that TCTP and phospo-TCTP may have functionally important interactions with microfilaments or microtubulis in a specific cell compartment. Further studies are necessary in order to clarify the implication of phospho-TCTP in cell shape regulation during carcinogenesis processes.

Consistent with the *in vitro* studies, we observed a clear association among phospho-TCTP levels, tumor grading and cell proliferation as indicated by the increase of Ki-67 expression. Moreover, the nuclear staining of phospho-TCTP was also shared by only a few cell clones in the primary tumor population. As tumors are characterized by an intratumor heterogeneity [49], the nuclear staining of phospho-TCTP may characterize a cell clone with a more aggressive phenotype and a survival advantage in the tumor cell compartment. Notably, we found an increase of phospho-TCTP levels in trastuzumab resistant tumors, indicating that the level of phospho-TCTP expression is correlated with enhanced tumor aggressiveness.

Our data are supported by findings of Desmedt et al. showing that the majority of genes overexpressed in high grade breast cancers are associated with cell proliferation and cell cycle progression [50].

Finally, we provide evidence that DHA in combination with selected cytotoxic agents, i.e. Doxorubicin for triple negative and Trastuzumab for HER+2 positive cancers, may provide an effective alternative to inhibit the progression of breast cancer cells by regulating the phospho-TCTP function. Our results also suggest the possibility of lowering chemotherapeutic doses, in combinatorial approaches, and hence, reducing side effects caused by anti-cancer drugs.

In summary, this study demonstrates that nuclear overexpression of phospho-TCTP is correlated with aggressive G2–G3 tumors and suggests that phospho-TCTP may be a prognostic marker and a therapeutic target. The combinatorial approach of DHA with anti-tumor drugs suggests a novel strategy to target advanced breast cancer.

## MATERIALS AND METHODS

### Chemicals

Dihydroartemisinin were from Selleck, BI2536 from Selleck, Paclitaxel from Fidia Farmaceutici; Doxorubicin from Fidia Farmaceutici; Cisplatin/cis-Platinum (II)Diammine Dichloride from Sigma.

Trastuzumab (Herceptin) was provided by Genentech (South San Francisco, CA).

### Antibodies

Antibody sources were as follows: anti-HRF/TCTP (MBL); anti-Phospho-TCTP (Ser46) (Cell Signaling); anti-c-Myc (Cell Signaling); anti-PARP (Cell Signaling); anti-HER2/ErbB2 (Cell Signaling); anti-cleaved caspase 3 (Cell Signaling); anti-Ub (P4D1); anti-β-actin (Sigma-Aldrich); anti-Lamin A/C (BD Biosciences); anti-α-Tubulin (Sigma-Aldrich).

### Cell culture and treatments

MDA-MB-231 (hereafter called MDA) (ER^−^, Pr^−^, Her2^−^), MCF7 (ER^+^, PR^+^, Her2^−^), SKBR3 (ER^−^, Pr^−^, Her2^+^), BT-474 (ER^+^, Pr^+/−^, /Her2^+^) cell lines were from the ICLC (Interlab Cell Line Collection, Genoa, Italy). HeLa cell line was from ATCC. All cell lines were maintained in RPMI-1640 or DMEM supplemented with L-glutamine, antibiotics and 10% heat-inactivated fetal bovine serum (FBS, all from Sigma-Aldrich) according to the ICLC indications. MCF 10A cell line was from ATCC. Cells maintained in Dulbecco's modified Eagle's medium (DMEM)/F-12 medium containing 5% horse serum, hydrocortisone (0.5 μg/ml), insulin (10 μg/ml), EGF (20 ng/ml), cholera toxin (100 ng/ml), penicillin 100 units/ml) and streptomycin (100 lg/ml) at 37°C in a 5% CO2 humidified atmosphere. All cell lines from ICLC or ATCC were characterized by DNA (SRT) profiling. Cells were immediately expanded and frozen such that they could be revived every 3 to 4 months.

Cells were cultured in a humidified incubator in an atmosphere of 5% CO2 at 37°C. Before any experiment, cells were detached by mild trypsinization, washed, plated in complete medium and allowed to recover for 24 h.

Cells were treated with DHA (Sellekchem) or BI 2536 (Selleckchem) dissolved in DMSO and diluted in culture medium immediately before use. Control media contained the same amount of DMSO-vehicle (<0.1%).

### Cell Viability assays

#### Proliferation assay

Exponentially growing cells (12 × 104 /plate) cells were seeded in 60 × 5 mm plates in complete medium. At the indicated times cells were detached with trypsin/EDTA and collected. Viable cells were incubated with Trypan blue and were counted with a haemocytometer.

#### ATP-assay

Exponentially growing cells (4 × 10^3^ /well) were plated in triplicate in 96-well dishes. Following treatments, the ATP content was determined luminometrically by the CellTiter-Glo Luminescent Cell Viability assay, following the instructions of the manufacturers (Promega). Luminescence was measured with an automatic microtiter plate reader, Victor 3 V, Wallac 1420, Multilabel Counter.

#### Caspase-3/7 activity assay

Exponentially growing cells (4 × 10^3^ /well) were plated in triplicate in 96-well dishes. Following treatments, cells were subjected to Caspase-3/7 activity measurement with Caspase-Glo 3/7 Assay (Promega). Briefly, cells were incubated in a cell lysis solution containing a luciferase substrate derivative, Ultra-GloTM Recombinant Luciferase for 1 hour at room temperature. The luciferase substrate derivative is specifically cleaved by active caspase-3/7 resulting in the conversion of the substrate into a luminescent signal (RLU). The luminescent signal was then normalized to cell number. Luminescence was measured with an automatic microtiter plate reader, Victor 3 V, Wallac 1420, Multilabel Counter.

### Western blot analysis

Cells were washed with ice-cold PBS, lysed in buffer contained 50 mM TRIS-HCl, pH 7.5, 150 mM NaCl, 1% Tryton X-100 (Sigma), 10 mM EDTA, pH 7.2, supplemented with a protease inhibitor cocktail (P1860-Sigma). Aliquots (10–40 μg) from total cell lysate proteins were resolved on 8% or 12% SDS–PAGE gels and analyzed by immunoBlotting with the indicated antibodies followed by decoration with peroxidase-labeled anti-rabbit (Thermo Scientific) or anti-mouse IgG (Dako) respectively. Blots were developed with ECL-plus (GE Healthcare), following the instructions of the manufacturer.

### Immunofluorescence

Cells were grown in coverslips fixed in 4% paraformaldehyde solution for 30 min and washed three times with PBS. Then, samples were permeabilized in 0.1% Triton X-100-PBS for 10 min. After blocking with 1% BSA, fixed cells were incubated with anti TCTP or anti-phospho TCTP antibodies for 1 hour and incubated with secondary antibody conjugated with Alexa Fluor 488 (Molecular Probes). Images were acquired on LEICA TCS SP5 Confocal Laser Scanning Microscopy (CLSM, Leica Instruments, Mannheim, Germany).

### Real-time PCR analysis

For real-time PCR analysis, total RNA was prepared using TRIzol and reversed transcribed using SuperScript reverse transcriptase (Invitrogen) following the instructions of the manufacturer. Real-time RT-PCR analysis was performed with ABI PRISM 7000 (Applied Biosystem), using RealMasterMix ROX (Eppendorf) to prepare the reaction mixes.

Primers for real-time PCR were the following:

TCTP-forward: 5′-TGAAGAACAGAGACCAGAAAG-3′

TCTP-reverse: 5′-CACGGTAGTCCAATAGAGCAAC-3′

GAPDH-forward: 5′-GAAGGTGAAGGTCGGAGTC-3′

GAPDH-reverse: 5′CATGGGTGGAATCATATTGGAA-3′

Actin-forward: 5′-GCGCTCAGGAGGAGCAAT-3′

Actin-reverse: 5′-CACTCTTCCAGCCTTCC-3′.

### Immunoprecipitation

Immunoprecipitations were done using 1 mg of protein from total cell lysates and 10 μL of polyclonal anti-ubiquitin antibody by incubating overnight at 4°C. The immune complexes were precipitated with Protein A-Agarose (Roche). Immunoprecipitates were resolved on 10% SDS-PAGE followed by Western Blotting with anti-TCTP antibody.

### siRNA transfection using lipofectamine RNAiMAX

MDA-MB-231 cells were plated and grown to 70% confluence at 24 h. Two hours prior to transfection the medium was replaced with 1X Opti-MEM reduced serum medium (Gibco).

ON-TARGET plus Human TPT1 siRNA-smart pool (Dharmacon) and ON-TARGET plus Non-targeting pool (Dharmacon) were transfected according to the Invitrogen protocol using Lipofectamine RNAiMax reagent (Invitrogen).

### Vector construction

Plasmid pcDNA3-TCTP was constructed by subcloning the BamHl-HindIII fragment of HrHRF/TCTP-pRSET A plasmid containing the human TCTP ORF into the same sites in pcDNA3.1(−)vector. To generate the N- Flag-tagged pcDNA3 vector two complementary oligos that introduce a FLAG cassette were annealed and ligated into Xhol-BamHl -cut pcDNA3 vector.

Fw: Xho-N-FLAG 5′ TCGAGATGGACTACAA AGACGATGACGACAAGG 3′

Rev: Bam-N-FLAG 5′GATCCCTTGTCGTCAT CGTCTTTGTAGTCCATC 3′

FLAG-TCTP-pcDNA3 was obtained subcloning the fragment BamHl-HindIII from pcDNA3-TCTP into the BamHl-HindIII-cut FLAG-pcDNA3 vector.

### Mutagenesis

Mutagenesis of sites of phosphorylation of TCTP Ser46 and Ser64 was performed with QuikChange site directed mutagenesis kit (Stratagene) with the following oligos obtained from web-based program PrimerX (bioinformatics.org/primerx):

S46

Fw: 5′ CAGAAGGTAACATTGATGACGCCCT CATTGGTGGAAATGCCTCC 3′

Rev: 5′ GGAGGCATTTCCACCAATGAGGGC GTCATCAATGTTACCTTCTG 3′

S64

Fw: 5′ CGAGGGCGAAGGTACCGAAGCCACA GTAATCACTGGTGTCG 3′

Rev: 5′ CGACACCAGTGATTACTGTGGCTTC GGTACCTTCGCCCTCG 3′

Oligos were all synthesized by PRIMM s.r.l. (Milan, Italy). Oligos for mutagenesis were used to mutagenize FLAG-TCTP -pcDNA3.1 vector. All costructs were confirmed by DNA sequence analysis.

### Cell transfection

HeLa cell lines stably expressing TCTP/HRF shRNA Plasmid or control shRNA Plasmid (Santa Cruz Biotechnology) vectors were selected by using puromycin antibiotic (1 μg/ml) 72 h after transfection. TCTP-silenced cells were cultured in DMEM supplemented with puromycin dihydrochloride antibiotic, and named HeLa_ctrshRNA_, HeLa _TCTPshRNA_, respectively. TCTP silenced-cells were transiently transfected with a plasmid encoding Flag tagged wild-type TCTP (WT) or a mutant lacking two serine residues 46 and 64 (AA). Mock-transfected cells were used as control. All transfections were done using Lipofectamine Plus reagent (Invitrogen) according to the manufacturer's protocols.

### Evaluation of cell sensitivity to combined treatment

Exponentially growing cells (4 × 10^3^/well) were plated in triplicate in 96-well. Cells were treated with DHA (20 and 50 μM), and after 24 h cell were exposed to treatment with DOX (0.1, 1 and 5 μM), or Trastuzumab (50 and 100 μg/ml) alone or in combination at various ratio. Growth inhibition was calculated as the percentage of viable cells compared with untreated cells by ATP assay. The CompuSyn software program was used to calculated synergistic, additive or antagonistic effects as a non-constant ratio combination. The CompuSyn software is based on the Median-Effect Principle (Chou) and the Combination Index-Isobologram Theorem (Chou-Talalay).

The combination index (CI) indicates a quantitative measure of the degree of drug interaction in terms of synergistic (CI < 1), additive (CI = 1) or antagonistic effect (CI > 1).

### Patients

Eighty-five cases of breast cancer were retrieved from the files of the Department of Pathology, at the National Cancer Istitute in Milan, Italy. The cases were selected according the patient clinical stage, histologic grade, and ER, PgR HER2 status. Ki-67 stains were also available in all cases. Tumors were scored ER and PgR positive when ≥ 10% of the nuclei were positive. HER2 was evaluated as 0, 1+, 2+, or 3+ according to ASCO/CAP guidelines (2013). All 2+ cases were evaluated by chromogenic in situ hybridization (CISH) to measure HER2 amplification. The cut-off for a “high” Ki-67 labeling index was ≥ 20% of positive tumor nuclei. Stage was determined according to UICC criteria [51]. Table 1 lists the clinico-pathologic characteristics of patients. Before undergoing routine surgery, all patients signed an informed consent authorizing the Institute to utilize their removed biological tissues for research purposes. Institutional approval from our Ethics Committee was obtained for the conduct of the study.

### Immunohistochemistry and CISH

Immunohistochemistry was performed by the standard biotin–streptavidin-peroxidase method on 3 μm-thick formalin-fixed, paraffin-embedded tissue sections. After deparaffinization in xylene and rehydration in descending concentrations of alcohol, an antigen retrieval step was performed by autoclave in 10 mM citrate buffer (pH 6). Endogenous peroxidase was blocked by 3% hydrogen peroxide. After blocking with Peroxidase-Blocking reagent for 5 min, the sections were incubated with the diluted primary antibody testing for the following markers: ER and PgR expression using 1D5 and PgR636 monoclonal antibodies (Dako), respectively; tumor proliferation using MIB1 monoclonal antibody (DAKO); HER2 oncoprotein overexpression using HercepTest (Dako), and polyclonal antibody pTCTP (Cell Signaling). The immunoreactivity was detected by using LSAB+ Kit (Dako) in room temperature according to the manufacturer's instructions. The 3,3-diaminobenzidine (DAB) (Liquid DAB+; Dako) solution was used as a chromogen. Sections were lightly counterstained with hematoxylin. The section without primary antibody served as negative control. Phospho TCTP expression was evaluated in each case as ≤ 1%, 1%–3%, 4–9%, ≥ 10%, of the tumor cells expressed nuclear phospho-TCTP.

For CISH staining procedures, sections were deparaffinized and heated in a 1M NaCNS solution 95°C for 10 minutes. After two 5-minute washes at 4°C in distilled water, the slides were incubated with 3 to 10 μg/ml proteinase K (EC 3.4.21.64) (Sigma, St.Louis, MO) for 10 to 15 minutes at 37°C. The slides were then washed (twice at 5 minutes each, at 4°C) in distilled water, dehydrated with graded ethanols, and air dried. The digoxigenin-labeled HER2 probe (double-stranded) (Zymed Laboratories Inc.) was applied to the slides, covered with coverslips, and denatured at 96°C for 6 minutes on a heat block. Hybridization was performed overnight at 37°C in a humid chamber. The slides were then washed for 5 minutes with 0.5 x standard saline citrate at 75°C, followed by a brief rinsing in phosphate buffered saline/0.25% Tween20. Immunodetection was performed according to the manufacturer's instructions. Finally, sections were lightly counterstained with hematoxylin.

### Statistical analysis

All experiments were done at least three times unless otherwise indicated. The results are presented as means ± SD. One-way ANOVA followed by the Bonferroni's test using the PRISM GraphPad software was used in the analysis of three or more data sets. Differences were considered to be significant for *P* < 0.05 and highly significant for *P* < 0.01 and *P* < 0.001. Association between phospho-TCTP expression and clinico-pathological parameters in breast cancer patients was analysed using the Chi-square test.

## SUPPLEMENTARY FIGURES AND TABLES


