# Supplementary material for: Phospho-TCTP as a therapeutic target of dihydroartemisinin for aggressive breast cancer cells
Source: Oncotarget. 2015 Mar 12;6(7):5275–91. doi: 10.18632/oncotarget.2971 (PMC4467148; doi:10.18632/oncotarget.2971)
Supplement: Supplementary file 1 [file oncotarget-06-5275-s001.pdf]

SUPPLEMENTARY FIGURES AND TABLES

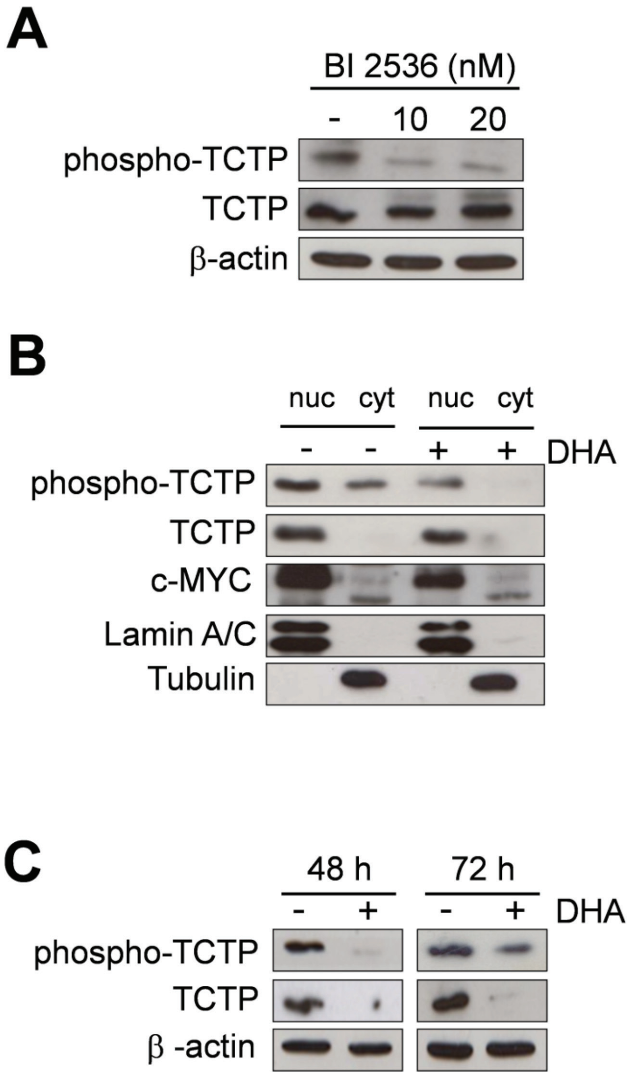

Supplementary Figure S1: Effect of DHA on phosphorylated TCTP levels. **(A)** Western Blot analysis of indicated proteins in SKBR3 cells treated with BI 2536 at indicated concentration for 48 h. β-actin was used as loading control. **(B)** Subcellular localization of indicated proteins in SKBR3 cells. Exponentially growing SKBR3 cells were treated with 50 μM DHA for 24 h. Nuclear and cytoplasmic fractions of untreated and treated cells were analyzed by Western Blot analysis. Lamin A/C and Tubulin were used as loading controls for nuclear and cytoplasmic fractions, respectively. **(C)** Western Blot analysis of indicated proteins in SKBR3 cells treated with 50 μM DHA for 48 h (upper panel) and 72 h (low panel). β-actin was used as loading control.

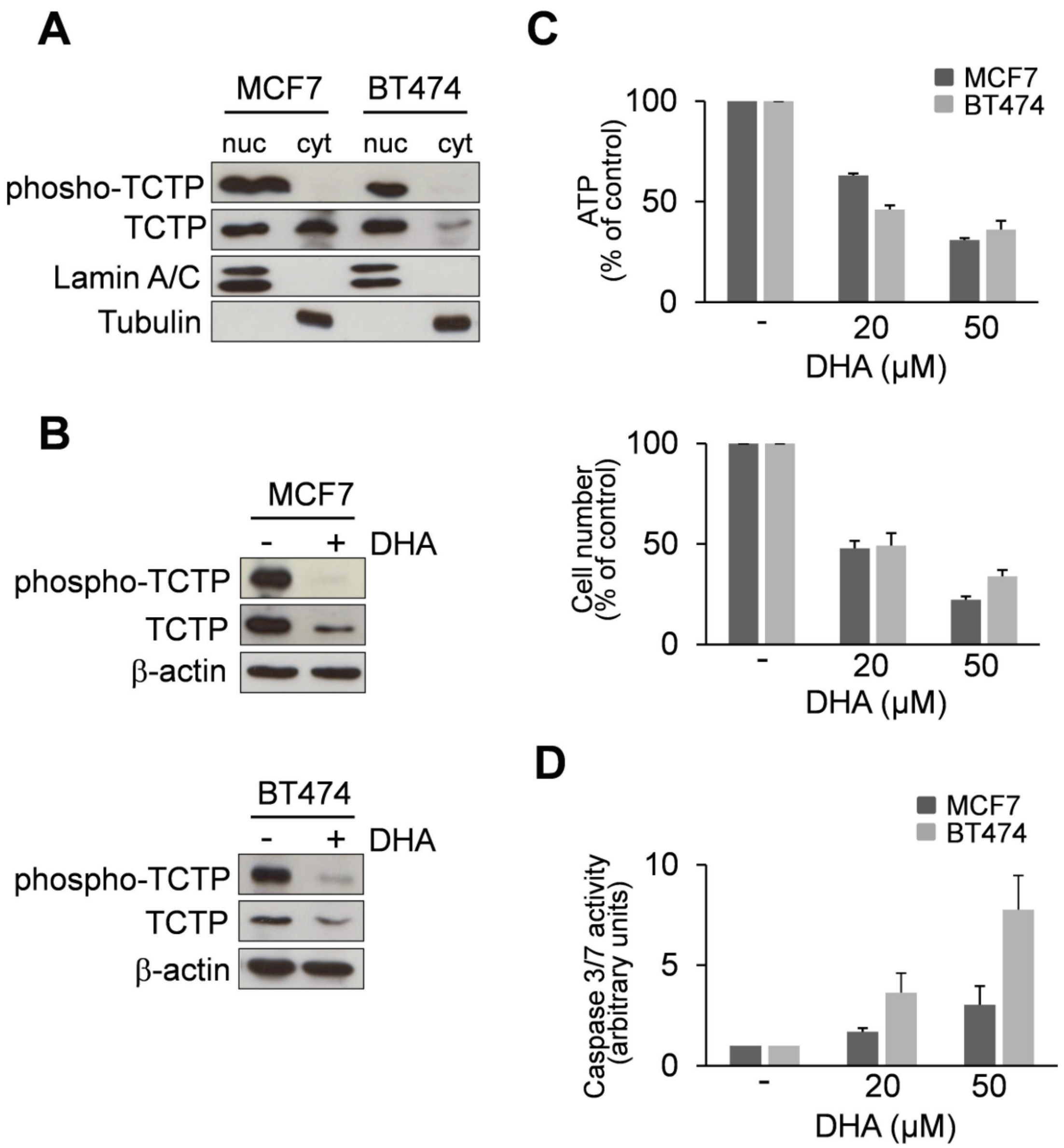

Supplementary Figure S2: Antitumor efficacy of DHA in MCF7 and BT474 cells. (A) Subcellular localization of indicated proteins in MCF7 and BT474 cells. Nuclear and cytoplasmic fractions of untreated and treated cells were analyzed by Western Blot analysis for the indicated proteins. Lamin A/C and Tubulin were used as loading controls for nuclear and cytoplasmic fractions, respectively. **(B)** Western Blot analysis of indicated proteins in MCF7 and BT474 cells after 72 h of exposition to DHA. β-actin was used as loading control. **(C)** MCF7 and BT474 cells were exposed to 20 μM, and 50 μM DHA for 72 h. Cell viability was measured by ATP assay (upper panel) and trypan blue dye esclusion assay (lower panel). Data are expressed as the percentage of viable cells relative to controls. Values represent the mean ± SD, $n = 3$. **(D)** Caspase-3/7 activity (normalized to cell number) was evaluated as a marker of apoptosis. Cells were treated as described in the legend 2C. Data are expressed as arbitrary units. Values represent the mean ± SD, $n = 3$.

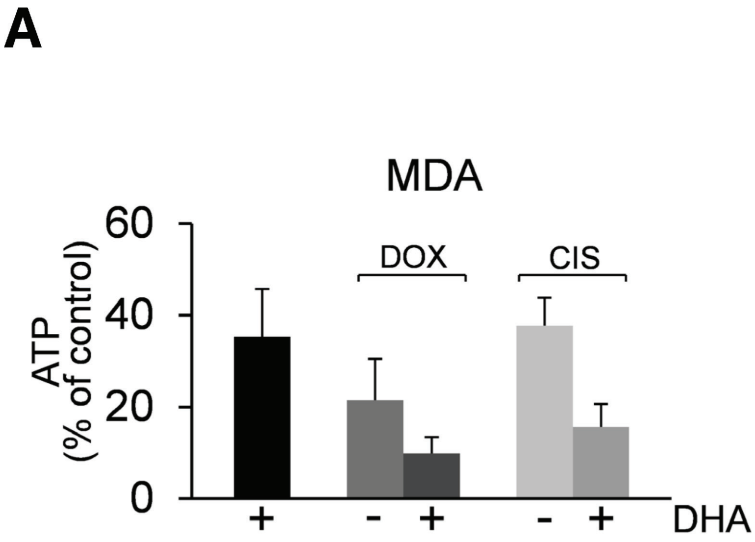

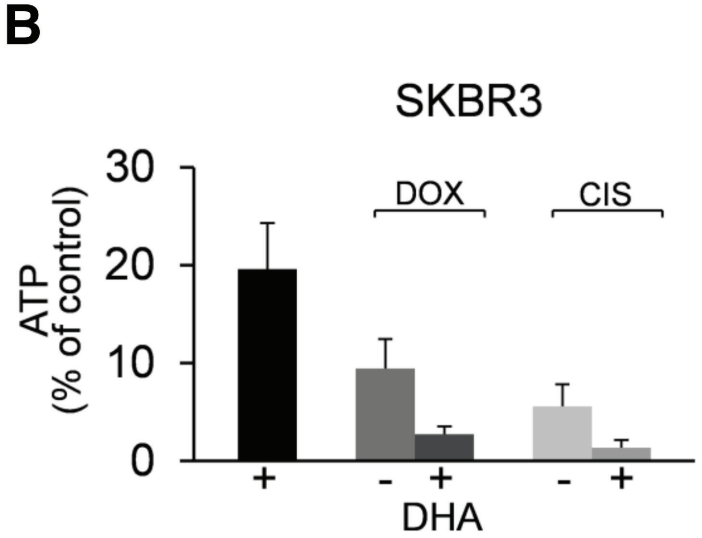

Supplementary Figure S3: Combination therapy of DHA with chemotherapy drugs. **(A)** MDA cells were treated with 50 µM DHA concentration for 24 h. Cell viability was measured after an additional 48 h treatment with 5 µM Doxorubicin (DOX) or 50 µM Cisplatin (CIS). At the end of incubation time, the number of viable cells was determined by ATP-assay. Data are expressed as the percentage of viable cells relative to controls. Values represent the mean ± SD, *n* = 3. **(B)** SKBR3 cells were treated with 50 µM DHA concentration for 24 h. Cell viability was measured after an additional 48 h treatment with 5 µM Doxorubicin (DOX) or 50 µM Cisplatin (CIS). At the end of incubation time, the number of viable cells was determined by ATP-assay. Data are expressed as the percentage of viable cells relative to controls. Values represent the mean ± SD, *n* = 3.

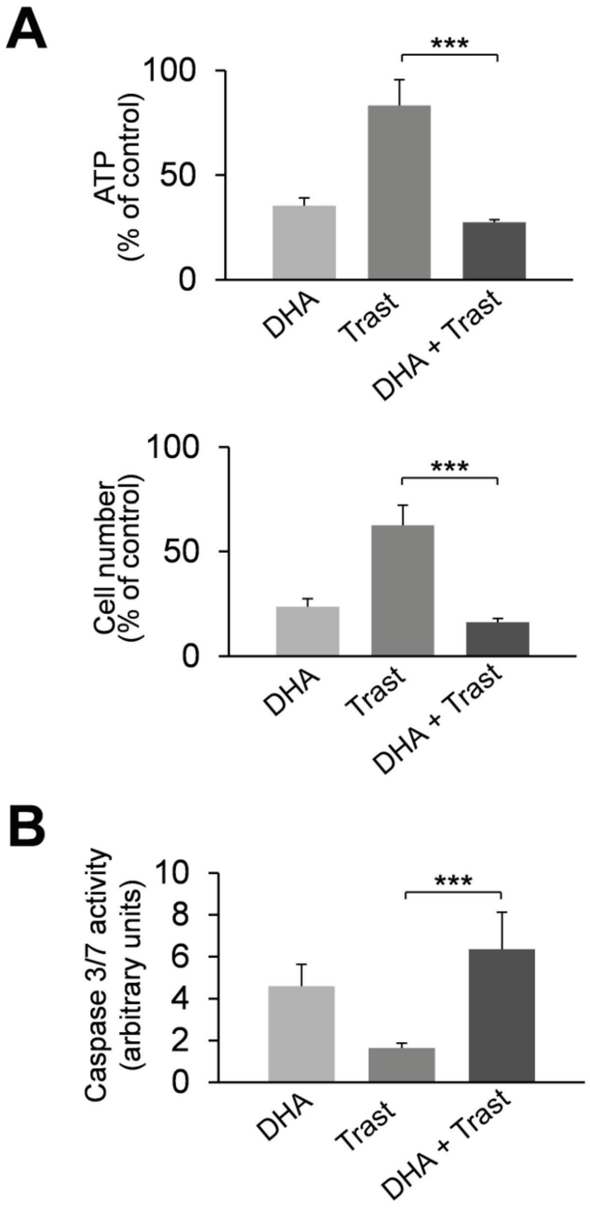

Supplementary Figure S4: Combination therapy of DHA with Trastuzumab. **(A)** SKBR3 cells were exposed to DHA (50 µM) and were then treated with 50 µg/ml of Trastuzumab (Trast) for 48 h. At the end of incubation time, the number of viable cells was determined by ATP-assay (upper panel) and by trypan blue dye exclusion assay (lower panel). Data are expressed as the percentage of viable cells relative to controls. Values represent the mean ± SD, $n = 4$. *** = $p < 0.001$. **(B)** Caspase-3/7 activity (normalized to cell number) was evaluated as a marker of apoptosis. Cells were treated as described in the legend S4A. Data are expressed as arbitrary units. Values represent the mean ± SD, $n = 4$. *** = $p < 0.001$.

Supplementary Table S1: DHA in combination with Doxorubicin causes synergistic inhibition of growth in MDA cells

DOXORUBICIN			
Drug		72 h	
DOX (µM)	DHA (µM)	Fractional inhibition	CI
$(D)_1$			
0.1		0,70	
1		0,52	
5		0,21	
	$(D)_2$		
	20	0,52	
	50	0,30	
$(D)_1 + (D)_2$			
1	20	0.22	0.419
1	50	0.19	0.661

The CalcuSyn software program was used to calculate synergistic, additive or antagonistic effects. Fractional inhibition = fraction decreased cell viability after treatment, control cells were set to “1”. CI = combination index. CI values below 0.9 indicate synergistic effect.

Supplementary Table S2: DHA in combination with Trastuzumab causes additive inhibition of growth in SKBR3 cells

Trastuzumab			
Drug		7 d	
Trast (µg/ml)	DHA (µM)	Fractional inhibition	CI
$(D)_1$			
100		0,44	
50		0,70	
	$(D)_2$		
	20	0,40	
	50	0,13	
$(D)_1 + (D)_2$			
50	20	0,29	1,070

The CalcuSyn software program was used to calculate synergistic, additive or antagonistic effects. Fractional inhibition = fraction decreased cell viability after treatment, control cells were set to “1”. CI = combination index. CI values = 1 indicate additive effect.
